# An uncommon case of postradiation atypical vascular lesions extending beyond the radiation field in a breast cancer patient

**DOI:** 10.1016/j.jdcr.2024.06.021

**Published:** 2024-07-06

**Authors:** Ying Sun, Emily Parks, Ahmed K. Alomari, Cameron Nichols

**Affiliations:** a Department of Pathology, East Carolina University, Greenville, North Carolina; b Department of Dermatology, East Carolina University, Greenville, North Carolina; c Department of Pathology, Indiana University, Bloomington, Indiana

**Keywords:** angiosarcoma, atypical vascular lesion (AVLs), breast cancer, post-radiation complication, radiation therapy

## Introduction

Atypical vascular lesions (AVLs) are vascular proliferations that rarely present following radiation treatment for breast cancer. The lesions typically develop within the field of radiation after a median of 3 years following radiation therapy.^1^^,^^2^ Histopathological features of postradiation AVLs can overlap with those seen in early well-differentiated radiation-induced angiosarcoma, especially in partial biopsies, posing diagnostic challenges.^3^ Postradiation AVLs are generally considered benign but can occasionally recur^4^ and rarely progress to angiosarcomas.^5^^,^^6^ Presently, local excision is the preferred treatment for solitary AVLs. In this report, we present an exceptional case of multiple postradiation AVLs involving skin outside the radiation field in a patient with a history of breast cancer and irradiation.

## Case report

**Fig 1:**
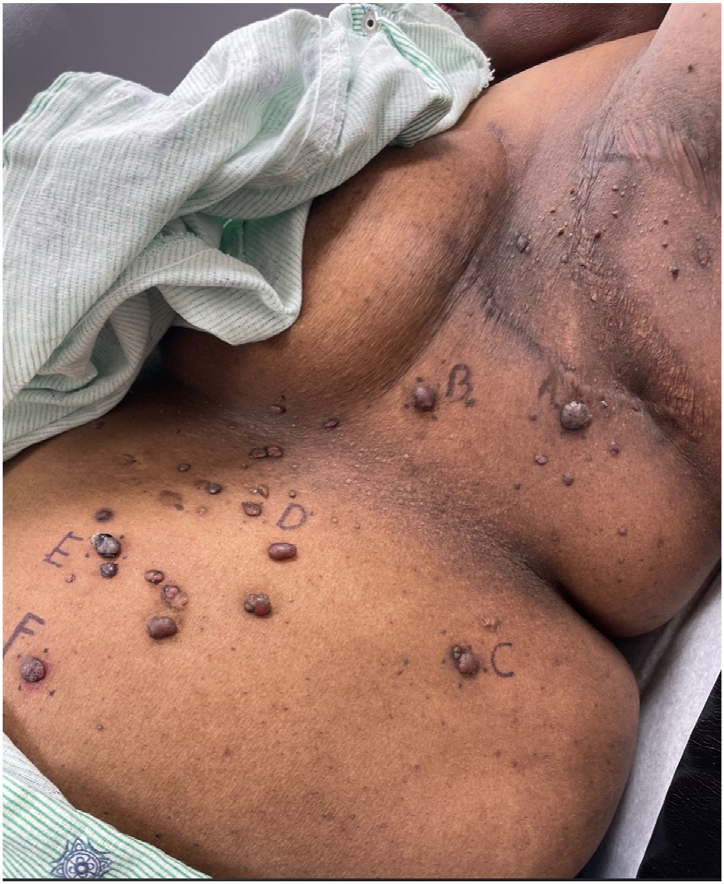
Multiple violaceous papules within the radiation field (left axilla) and extending outside the radiation field (left flank and anterior abdominal wall).

A 57-year-old African American female presented to clinic with a 3 month history of numerous pruritic, painful lesions on the left axilla and flank. The lesions were growing progressively larger and some of the lesions had bled. Physical examination was notable for more than 25 hyperkeratotic, violaceous papules on the left axilla, left flank, and anterior abdominal wall (Fig 1).

The patient’s medical history was notable for bilateral breast cancer status post right breast mastectomy and left breast lobectomy in addition to radiation therapy and hormonal therapy. Radiation therapy included a total dose of 60 grays in 30 fractions, which is consistent with conventional doses of radiation for breast cancer. At the time of presentation, the patient was 8 years post radiation therapy. The patient declined genetic testing following her breast cancer diagnosis. There were no signs of lymphedema on follow up examinations after radiation therapy.

**Fig 2:**
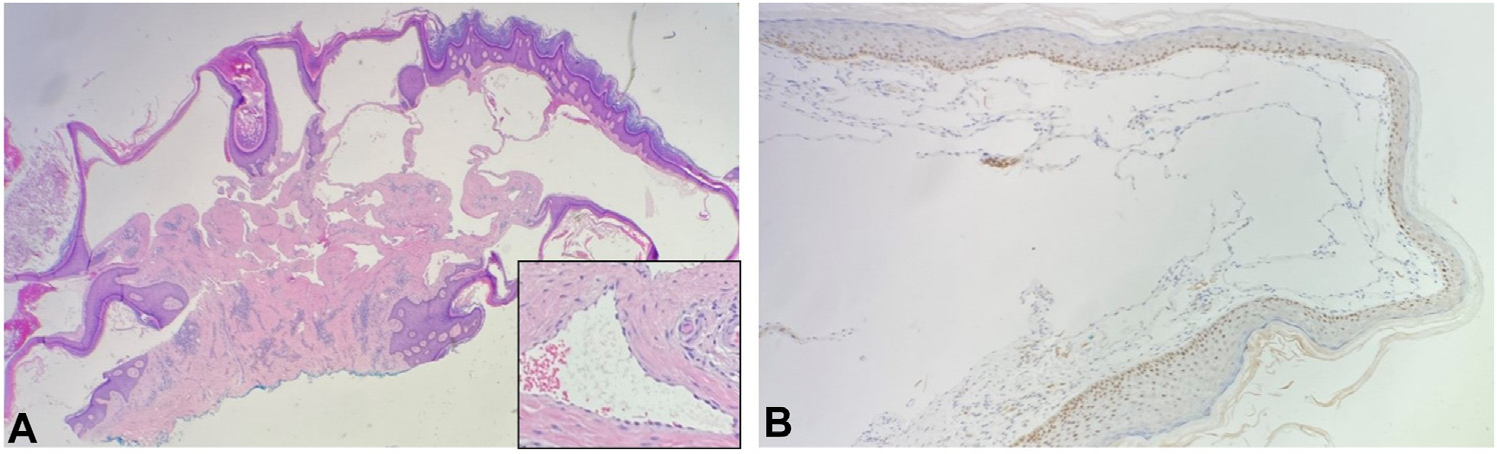
Histopathologic findings from initial biopsy of a post radiation atypical vascular lesion (AVL) in the left axilla. **A,** (Hematoxylin and eosin [H&E], × 20). Proliferation of markedly dilated, irregular, anastomosing, lymphatic-like vascular spaces in the superficial dermis, accompanied by epidermal collarette. **B,** Immunohistochemical stain for c-Myc negative in the endothelial cells within the AVLs, with a positive internal control.

**Fig 3:**
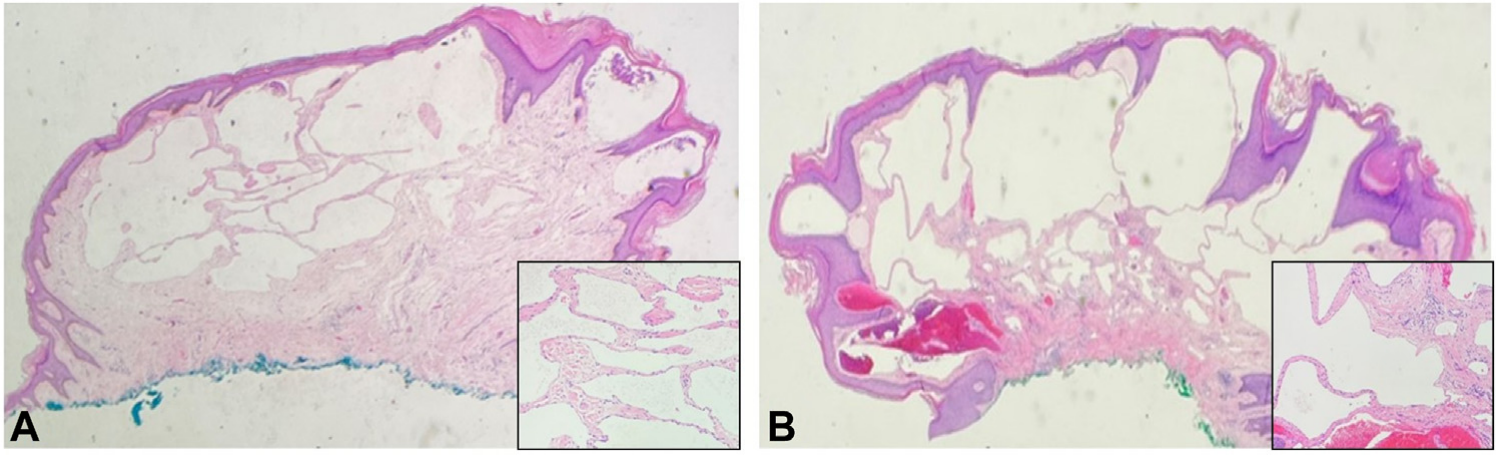
Histopathologic findings from subsequent biopsies of multiple atypical vascular lesions (AVLs). **A,** Left flank (Hematoxylin and eosin [H&E], × 20; H&E ×400) and (**B**): anterior abdominal wall (H&E, × 20; H&E ×400). Low power view reveals irregular, anastomosing, lymphatic-like vascular channels within the upper and mid dermis. Epidermal collarettes surround the lesions. The vascular channels appear dilated and exhibit a branching pattern. High power view demonstrates a single layer of endothelial cells within the vascular channels, absent of significant nuclear atypia.

A shave biopsy of a lesion in the left axilla was performed and revealed histopathological characteristics of post radiation AVLs, including markedly dilated, irregular, anastomosing, lymphatic-like spaces lined by a single layer of flattened-to-hobnail endothelial cells in the superficial dermis, accompanied by an epidermal collarette. There was no significant cytologic atypia, mitotic activity, or endothelial multilayering (Fig 2, *A*). Immunohistochemistry for c-Myc was negative (Fig 2, *B*). Subsequent biopsies of other lesions in the left axilla, left flank, and anterior abdominal wall showed similar histological features (Fig 3, *A* and *B*), consistent with a diagnosis of multiple postradiation AVLs. None of these biopsies showed overt malignant features.

Local excision is the standard of care for AVLs and staged excision of the lesions was discussed, however the patient elected to proceed with close clinical monitoring without excision of remaining lesions. The patient remained stable without any new or changing lesions until 12 months following the initial biopsy, at which time she reported a new lesion to the left inframammary region.

## Discussion

Radiation therapy is a cornerstone in the treatment of breast cancer, though carries the potential for rare complications such as AVLs. Postradiation AVLs were first described in mammary skin following radiation therapy for breast carcinoma in 1994 and typically remain confined to the radiation field.^7^ The development of AVLs outside the radiation field is exceedingly rare, with only 2 reported cases in the existing literature.^8^^,^^9^ One remarkable aspect of our case is the extensive involvement of AVLs outside the radiation field.

Post radiation AVLs can often be challenging to diagnose, as their histological features can overlap with angiosarcoma, especially in small partial samples.^2^^,^^3^ In this case, a malignant vascular process was suspected clinically given the multifocal involvement of skin outside the radiation field. Nevertheless, histopathological examination of several of these lesions failed to show features of malignancy or overexpression of c-Myc by immunohistochemistry typically observed in radiation-induced angiosarcoma.^10^ The histopathological and immunohistochemical findings in this case align with the typical characteristics seen in post radiation AVLs.

Although post radiation AVLs are generally considered at the benign end of the spectrum of radiation-induced vascular proliferations, the possibility of progression to malignant angiosarcoma remains a concern. Such progression, though rare, has been documented in the literature and might be of increased likelihood in patients with several AVLs.^5^^,^^6^ Moreover, the mechanism of the development of AVLs outside the radiation field remains unclear and it is unknown whether such presentations might be associated with faster or greater likelihood of evolution to angiosarcoma. Established treatment guidelines for such cases are lacking. In this case, the discussion of staged excision was appropriate given the standard management of solitary AVLs and the uncertainties regarding this presentation. Staged excision would permit the gradual removal of multiple lesions, examination of the entire border and thus minimize the risk of recurrence and progression while preserving as much healthy tissue as possible. However, the extensive area of involvement presented a therapeutic challenge that may result in significant morbidity with multiple surgeries; therefore, the shared decision to proceed with close clinical monitoring thus far proves to be a reasonable approach. The uncertainty surrounding the long-term prognosis and risk of progression to malignant angiosarcoma in cases of extensive AVLs outside the radiation highlights the need for close clinical follow-up and ongoing surveillance.

## Conclusion

This case report sheds light on an unusual presentation of post radiation AVLs extending beyond the radiation field in a breast cancer patient, manifesting 8 years after radiation therapy. It presents unique challenges in terms of successful diagnosis and management. While AVLs are generally considered benign, the potential for progression to malignant angiosarcoma in such unique presentation remains uncertain. Meticulous histopathological examination and careful consideration of treatment options are essential. Further research and documentation of similar cases will enhance our understanding of this rare postirradiation treatment complication and ultimately guide clinical management.

## Conflicts of interest

None disclosed.
